# Competency in invasion science: addressing stagnation challenges by promoting innovation and creative thinking

**DOI:** 10.1007/s00267-024-02035-8

**Published:** 2024-09-05

**Authors:** Phillip J. Haubrock, Irmak Kurtul, Rafael L. Macêdo, Stefano Mammola, Ana Clara S. Franco, Ismael Soto

**Affiliations:** 1 Department of River Ecology and Conservation, Senckenberg Research Institute and Natural History Museum, Frankfurt, Gelnhausen, Germany; 2 Faculty of Fisheries and Protection of Waters, South Bohemian Research Centre of Aquaculture and Biodiversity of Hydrocenoses, University of South Bohemia in České Budějovice, Zátiší 728/II, 389 25 Vodňany, Czech Republic; 3 CAMB, Center for Applied Mathematics and Bioinformatics, Gulf University for Science and Technology, Al-Abdullah, Kuwait; 4 Marine and Inland Waters Sciences and Technology Department, Faculty of Fisheries, Ege University, İzmir, Türkiye; 5 Department of Life and Environmental Sciences, Faculty of Science and Technology, Bournemouth University, Poole, Dorset, UK; 6 Institute of Biology, Freie Universität Berlin, Königin-Luise-Str. 1-3, 14195, Berlin, Germany; 7 Leibniz Institute of Freshwater Ecology and Inland Fisheries (IGB), Müggelseedamm 310, 12587, Berlin, Germany; 8 Graduate Program in Ecology and Natural Resources, Department of Ecology and Evolutionary Biology, Federal University of São Carlos, UFSCar, São Carlos, Brazil; 9 Molecular Ecology Group (MEG), Water Research Institute (IRSA), National Research Council (CNR), Corso Tonolli, 50, Verbania, 28922 Italy; 10 Finnish Museum of Natural History (LUOMUS), University of Helsinki, Pohjoinen Rautatiekatu 13, Helsinki, 00100 Finland; 11 NBFC, National Biodiversity Future Center, Palermo, 90133 Italy; 12 Institute of Aquatic Ecology, University of Girona, 17003 Girona, Catalonia Spain

**Keywords:** Competence, Novelty, Evolution, Invasion science, Innovation, Transdisciplinarity

## Abstract

In today’s ever-evolving scientific landscape, invasion science faces a plethora of challenges, such as terminological inconsistency and the rapidly growing literature corpus with few or incomplete syntheses of knowledge, which may be perceived as a stagnation in scientific progress. We explore the concept of ‘competency’, which is extensively debated across disciplines such as psychology, philosophy, and linguistics. Traditionally, it is associated with attributes that enable superior performance and continuous ingenuity. We propose that the concept of competency can be applied to invasion science as the ability to creatively and critically engage with global challenges. For example, competency may help develop innovative strategies for understanding and managing the multifaceted, unprecedented challenges posed by the spread and impacts of non-native species, as well as identifying novel avenues of inquiry for management. Despite notable advancements and the exponential increase in scholarly publications, invasion science still encounters obstacles such as insufficient interdisciplinary collaboration paralleled by a lack of groundbreaking or actionable scientific advancements. To enhance competency in invasion science, a paradigm shift is needed. This shift entails fostering interdisciplinary collaboration, nurturing creative and critical thinking, and establishing a stable and supportive environment for early career researchers, thereby promoting the emergence of competency and innovation. Embracing perspectives from practitioners and decision makers, alongside diverse disciplines beyond traditional ecological frameworks, can further add novel insights and innovative methodologies into invasion science. Invasion science must also address the ethical implications of its practices and engage the public in awareness and education programs. Such initiatives can encourage a more holistic understanding of invasions, attracting and cultivating competent minds capable of thinking beyond conventional paradigms and contributing to the advancement of the field in a rapidly changing world.

## Introduction

Throughout history, cultural development has thrived in both times of prosperity and periods of adversity, driven by the dynamic interplay of human creativity and the material conditions of life, reflecting deliberate innovations and adaptive responses to socio-economic challenges (Landry 2012; Sawyer and Henriksen 2024). Williams (1958) argues that these cultural shifts are part of the continuous process of defining and redefining our collective way of life, influenced by both dominant and emergent social structures. This process is also critical in understanding the role of culture in perpetuating and challenging existing power dynamics (Horkheimer and Adorno 1944). Nowadays, however, the vast scope of global change and the challenges of international interconnectedness often blur the lines between cultural stagnation and evolution (Lull 2008). This ambiguity makes it challenging to discern whether cultural shifts are substantial transformations of societal values and practices or merely superficial changes. In today’s interconnected world marked by unique challenges and opportunities, cultural development is no longer a straightforward journey echoing the past. Instead, it has become a multifaceted process deeply influenced by global interconnectedness and the rapid pace of technological advancements. This transformation demands a specific set of competencies, especially the ability to produce actionable science that supports real-world decision-making in areas such as climate change and environmental management (Goolsby et al. 2023), including the ability to evaluate ecological data within the context of societal values and conservation goals (Heger et al. 2019).

## The concepts of competence and competency

The concept of competence has a controversial and long history of technical significance compared to its use in the fields of psychology and linguistics, tracing back to Roman law, where this concept held importance in jurisprudence (Fügemann 2004). Competence in science has been defined and applied to different fields, seeking to clarify the concept within a holistic competence typology (Le Deist and Winterton 2005). This has led to the understanding of the term ‘competence’ varying depending on its intended usage, contributing to the perspective that establishing a universally valid definition of competence meeting scientific criteria is an unattainable objective (Stoof et al. 2002; Hartig 2008). Nevertheless, definitions have been applied, such as in the field of psychology, where competence commonly refers to a demonstrable behaviour (Moore et al. 2002). Revealing a lack of consensus on the nature of definitions, concepts, and their components underlying competence and competency, Fernandez et al. (2012), Blömeke et al. (2015) and Jacques (2016) describe competence as a process (referring to a directed event). In contrast, Short (1985) suggested that competence is not static but influenced by external circumstances, in opposition to personal qualities, which remain stable over time, Carrier and Wimmer (1995) describe competence as a continuum (implying cohesion and continuity). This view is complemented by Schneider (2019), who emphasized the importance of these stable qualities over situational competence in determining the overall quality of performance. To date, however, the concept of competencey is primarily applied in the fields of psychology and education (Salman et al. 2020), where it has been extensively studied and discussed (Dzhengiz and Niesten 2020) and extended (Ovbiagbonhia et al. 2019). Contrary to the widespread belief, ‘competence’ merely refers to specific skills or proficiencies (often seen as a combination of skills, knowledge, and behaviors), whereas ‘competency’ as a related concept signifies a broad capacity for effective action through skills, knowledge, and behaviours, including the ability to inquire, innovate, and address challenges. Competency is therefore defined as an ability to (self-)improve beyond mere technical skills or proficiencies to include a broader set of (continuously developed) personal capabilities that can be enhanced through experience and learning to facilitate *superior performance* (i.e., competence; Boyatzis 1982, 2008). Although the analysis of competence concepts is, from a philosophical standpoint, impractical due to its inherently technical nature (Roeger 2016), competence may rather represent a mechanistic, technically-oriented way of thinking that is often inadequate for describing human actions or facilitating human training (Ashworth and Saxton 1990).

Competency, in this context also known as adaptive, creative, or innovative competence, however, extends beyond just having the knowledge or skills to address known problems or tasks; it also encompasses the capacity for innovation, problem-solving, and thinking ‘outside the box’ (not necessarily with preceding education on a subject; Calavia et al. 2021) as a requirement to implying an individual’s capability to go beyond applying existing knowledge and skills, describing the ability to answer previously unasked questions, think creatively, and innovate with novel and potentially disruptive ideas rethinking previous paradigms by generating new ideas, solutions, and approaches to unforeseen challenges (Ovbiagbonhia et al. 2019, Herberg and Torgersen 2021).

## Stalling progress, sparking innovation

Meanwhile, several recent studies suggest that scientific progress is slowing in several major fields (Pammolli et al. 2011; Bloom et al. 2020), with the total number of papers published in the field of life sciences increasing exponentially (Landhuis 2016; for example, >6.7 million new scientific papers entered the repository of scientific literature in 2022), while the relative number of highly disruptive papers (i.e. those that alter, challenge, or change existing norms, theories, methods, or practices within their respective fields) has decreased in relative terms (Park et al. 2023). While the number of published papers is steadily increasing and journal impact factors inflate, the competition for funding, positions, and other resources intensifies, exacerbated by their limited availability. Paired with a persistent need to secure funding, this could lead researchers to focus on research with guaranteed outcomes rather than innovative or disruptive ideas, which might be seen as riskier. A scarcity of funding, uncertainties regarding study outcomes and the pursuit of quantity over quality, exemplified by the preference for incremental progress rather than groundbreaking contributions (e.g. ‘salami slicing’), have led to a diminishing occurrence of long-term studies (Lindenmayer et al. 2012). This trend persists despite the considerable contributions of such studies in natural sciences (Strayer et al. 2006), deflecting attention from the essential goals of propositive and sustainable knowledge-based science aimed at preserving biodiversity and enhancing human well-being.

The *publish-or-perish* culture in academia might further incentivize quantity over quality. This has resulted in the replicability crisis in science (Manufò et al. 2017), marked by an increasing number of paper retractions, highlighting a systemic failure in promoting genuine scientific advancement and competency. This crisis is exacerbated by a publication culture prioritizing sensational headlines and novelty over rigorous, reproducible research. Such trends not only mislead early career scientists and underrepresented groups but can also undermine the integrity and relevance of scientific discourse, setting a precarious stage for the challenges described in the subsequent discussion. Thus, researchers may focus on publishing more papers to secure tenure or funding rather than taking the time to develop groundbreaking ideas, to the point of compensating the lack of originality with overly sensationalised language to oversell their results and increase the chances of publishing in higher impact journals (Sumner et al. 2014; Scott and Jones 2017; Mammola 2020). This is exemplified by an increasing number of researchers globally and, at the same time, a contraction of budgets from both public and private funding sources in recent years due to economic hardships, increasing even more the competition among researchers (Johnstone and Marucci 2007; Carlson et al. 2015; Mitchell et al. 2019; Alabdullah et al. 2020). Established senior researchers therefore often exploit young postdoctoral and early career researchers, frequently without reciprocation, and burden them with teaching and additional responsibilities (Jamali et al. 2020; Cohen and Baruch 2022). Additionally, the future prospects for these early career researchers to secure permanent contracts and stable work environments are bleak (Aarnikoivu et al. 2019). This is, however, not to say that only younger researchers may be at the losing end. Considering the higher number of researchers paired with a lack of permanent positions and a lack of safe working environments, numerous researchers are nowadays struggling (Dirnagl 2022). This problem is even exacerbated considering that early career researchers often have more access to scientific funding than their older counterparts, as if previous contributions are no longer important and the focus should solely be on future research (Kent et al. 2022; Tsugawa et al. 2022). Competition may become even more challenging for members of vulnerable groups, such as individuals with disabilities, gender disparities, researchers affiliated with smaller universities, or non-English native speakers with lower success rates in securing grants (Wahls 2016; Stadmark et al. 2020; Swenor et al. 2020). To foster disruptive ideas and innovation, there is a need for more funding bodies and specifically designated funding to increasingly target *high risk, high reward* fundamental research, which is not necessarily backed-up with preliminary data. This funding architecture can help to move novel ideas forward, particularly between distinct disciplines, while not neglecting empirical applications and impact or negating the steady progression of current research themes.

Despite the surge in the volume of published research and a growing trend towards collaborative team-based scientific endeavours and ongoing creative discourse (Yanai and Lercher 2024), the percentage of aspiring researchers who attain long-lasting careers in academia has decreased, while the population of temporary scientists has grown considerably (Milojević et al. 2018). Considering that productivity and the ability to ‘survive’ in academia seem to be linked, neither the level of productivity nor the citation impact of one’s initial work, nor the degree of initial collaboration, can reliably predict long-term success in the respective field (Milojević et al. 2018). Concomitantly, works of considerable significance are often only recognized after years or even decades (so-called ‘sleeping beauties’; Ke et al. 2015), suggesting an often-delayed recognition of both competence and especially competency. The occurrence of competency as the ability to notably advance the subject of study and our concomitant understanding is, however, a rare phenomenon that can occur temporarily when confronted with a specific issue (Fine 1991; Masten 2012) or persist when nurtured, underlining the importance of the development of competent minds as it demands a stable environment to be fostered, presenting the basis for the advance of scientific disciplines.

## Competency in invasion science

The term ‘biodiversity’ is frequently used in scientific literature, often to artificially enhance the appeal of research that might be limited in scope to specific taxonomic groups, habitats, or facets of biodiversity, such as taxonomic, (phylo-) genetic, or functional diversity. Mammola et al. (2023) emphasised that, despite advancements in analytical tools, monitoring technologies, and data availability, the taxonomic scope of research articles falsely labelled under the umbrella term (and ‘buzzword’) ‘biodiversity’ has not broadened in recent years. This situation mirrors the broader challenges within invasion science. The field of invasion science is a relatively young discipline (Latombe et al. 2019) that rapidly developed following the realisation of the increasing socio-economic, ecological, and thus systematic threat presented by the introduction of non-native species (Simberloff et al. 2005; Pyšek et al. 2020). Despite its youth, invasion science has made significant strides forward, driven by notable works that have illuminated several crucial aspects, including the (*i*) threats posed by biological invasions (Simberloff et al. 2013), (*ii*) conceptual schemes and intricate relationships between concepts such as ‘spread’ and ‘impact’ of invasive non-native species (Clowl et al. 2008; Blackburn et al. 2011; Soto et al. 2024), and, among many other relevant aspects, (*iii*) studies revealing the importance of different dispersal pathways and vectors facilitating regional or global spread (Hulme 2009).

As the body of literature on non-native species expands, an increasing number of studies present evidence and caution against the threats posed by them (Pyšek et al. 2020) while identifying potential areas of future significance (Ricciardi et al. 2021) and introducing frameworks for e.g. generalised impact assessment (Blackburn et al. 2011; Hawkins et al. 2015). Nowadays, researchers are caught in a repetitive cycle of publishing paper after paper, meticulously charting the impacts of biological invasions year by year. This relentless focus on specific impacts yields valuable local insights but falls short of providing a general understanding or solution to the broader issue of biological invasions. Yet, according to the Scientific Committee on Problems of the Environment (SCOPE), the main challenges remain barely studied, such as (1) *What factors determine whether a species becomes invasive?* (2) *What attributes determine if an ecosystem is resilient or susceptible to invasions?* or (3) *How could invasions be managed?* (Simberloff, 2011) the same that are crucial for tackling future challenges. At the same time, there is a positive feedback loop of research towards well-known invaders while most of the species lack any data.

Despite numerous studies, the underlying problems of increasing introduction rates and established (or even spreading) populations of harmful non-native species persists, indicating a need for more comprehensive and effective management approaches. However, amidst the proliferation of publications in invasion science, a notable portion may appear to offer only marginal contributions to existing knowledge. Notably, Hulme and Mclaren-Swift (2022) highlight the importance of effective communication in the field (i.e. to stakeholders, citizens, etc.) e.g. through improved readability of studies through advancements in data visualisation, knowledge synthesis (Richardson et al. 2011; Jeschke et al. 2021; Ahmed et al. 2023) and the adoption of cutting-edge strategies like machine learning. Measuring the relevance and impact of papers in the field of invasion science however remains difficult. The number of papers and citations are classic examples, yet while papers that enhance our knowledge and understanding are important, those that lead to policy change or offer valuable solutions ultimately remain extremely rare also due to the lack of consensus in the field (Simberloff et al. 2024; Soto et al. 2024). Also, while drawing increased attention to the issues associated with biological invasions, some recently published and highly cited works predominantly emphasise already widely acknowledged information (Pyšek et al. 2020), not previously demonstrated, yet often used as premise. This may suggest that invasion science itself has become an increasingly closed system perpetuating numerous works not based on merit but the individual author’s connections (Boyer 2003; Gazni and Thelwall 2014; Zhang et al. 2021). Beyond the overarching debates surrounding ‘denialism’, which refutes the evidence for negative impacts posed by biological invasions (Ricciardi and Ryan 2018; Cuthbert et al. 2020; Linklater et al. 2023), these debates may promote critical self-reflection and innovative thinking (Chen et al. 2022), thereby promote civilised discussions that ultimately lead to new schools of thinking. However, some critics may contend that any scientific discipline will slow down over time. A slowdown in scientific progress and disruptive advancements (which would require formulating new ideas and testing them) could indicate several possibilities: either all relevant research has been conducted, all worthwhile questions have been answered, or more likely, unexplored questions have not been asked or investigations remain, but the necessary data may not be currently available. Hence, competency in invasion science would encapsulate the wide-ranging ability of invasion scientists to not only effectively address and tackle challenges posed by non-native species but also to innovate invasion science as a discipline by identifying current challenges (such as issues arising prioritisation and management; Gherardi et al. 2011) and major challenges that may arise in the future (such as the dualism of non-native species impacts; Gbedomon et al. 2020), the standardisation of terminology (Soto et al. 2024), and the relationship of rewilding and biological invasions to prioritise in invasion science (Ricciardi et al. 2021).

Despite the accelerated development, one core focus of invasion science—predicting which species will exert impacts elsewhere or quantifying invasion impacts in general—remains difficult or even stagnant. This stagnation is due to the variable nature of cultural value systems and a predominant focus on biological and ecological aspects over management strategies such as public education and policymaking. Even the recent quantifications of impacts in monetary terms (Diagne et al. 2021), which holds the merit of making the concept of biological invasions more concrete and accessible to the general public, stakeholders, and policymakers (Fantle-Lepczyk et al. 2022), remains incomplete as socio-cultural impacts remain difficult to quantify in e.g., monetary terms (Bacher et al. 2018; Jaríc et al. 2021). Moreover, while invasion science has grown as a discipline and made substantial progress, scientists have fallen into several fallacies. These included, for instance, the erroneous classification of merely reported species as established, misleading analyses using cumulative numbers, or the use of impact to define invasiveness, but also the generalisation of findings at the species level, overlooking the sometimes subtle but often considerable differences between populations (Haubrock et al. 2024). However, as Soto et al. (2024) noted, the rapid advance of this field has led to a proliferation of polysemous terminology. Even though nuanced and precise terminology is crucial for accurately interpreting and applying research findings in invasion science (and other related disciplines, e.g., biodiversity studies), the inaccurate application of terms could also alleviate misunderstandings. This ultimately also resulted in the use of ambiguous terminology and misclassifications of entire species as invasive by governments and stakeholders (and as such, the creation of deny lists; Essl et al. 2011) and an overemphasis of an invader’s impacts rather than the more relevant ability to spread (Soto et al. 2024). Shortcomings like these, paired with the toxic environment for young minds trying to find a safe and permanent home in science (Müller 2014; Boudia and Jas 2022; Bloch et al. 2023), hinder the development of invasion science as a field and deter competent minds.

In a recently published study, several highly respected invasion scientists published a dire warning about the threat posed by invasive non-native species entitled ‘Scientists’ warning on invasive alien species’ (Pyšek et al. 2020). This highly cited and go-to reference effectively summarises the current state of the art about non-native species, highlighting five research priorities. Similarly, to advance invasion science in the face of environmental change Ricciardi et al. (2021) proposed four core priority areas for future invasion scientists, namely (1) the development of a comprehensive framework for predicting non-native species behaviour and their ecological impacts in different environments, (2) an investigation of how climate change and multiple stressors affect the establishment of non-native species and adapt management strategy, (3), addressing the shortage of taxonomic expertise to enhance the detection and assessment of invasion risks, and (4) considering the global dispersal networks’ bridgehead effects in international biosecurity strategies. Despite not providing any feasible solution to any of the key issues surrounding biological invasions, finding solutions is arguably integral to the well-being of humans. However, addressing the proposed priority areas seems unfeasible given global disparities in addressing biological invasions (Nuñez et al. 2022), disparities in classification (Haubrock et al. 2023) and terminology (Soto et al. 2024), and the unpredictable nature and effects of climatic changes (Roe and Baker 2007; Pecl et al. 2017; Le Lann et al. 2021), or anthropogenically facilitated environmental change (Vitousek et al. 1996; Tuomainen and Candolin 2011), among other factors.

**Fig. 1:**
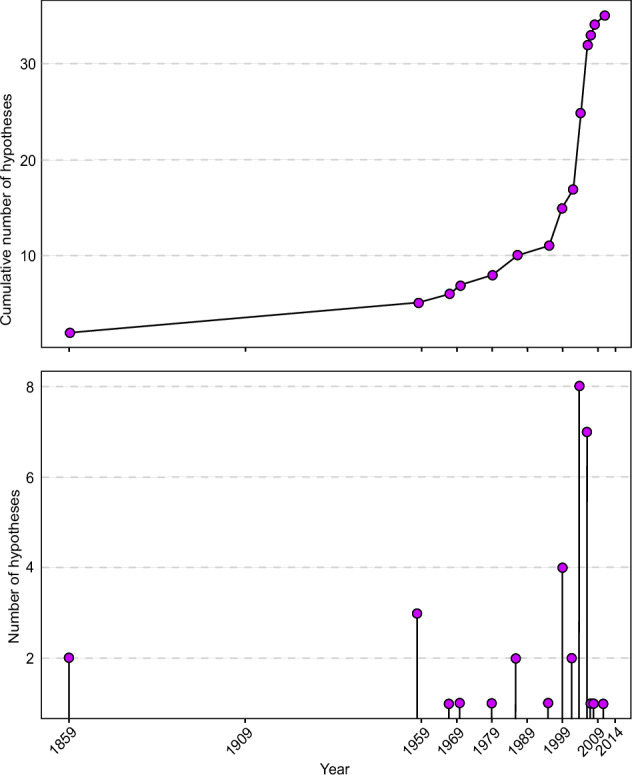
Temporal trend of hypotheses published in the field of invasion science plotted both as raw annual values (**a**) and (**b**) cumulatively according to Daly et al. (2023)

Competency in invasion science can be defined as the ability to advance the field through creatively and critically approaching ecological challenges, questioning established theories, developing innovative strategies for managing and understanding biological invasions and invasion dynamics, and identifying the questions worth asking to explore new avenues of inquiry and to adapt to a constantly changing world. Thus, to better illustrate the slower ratio of discovery of novel ideas and disruptive advances in the invasion sciences field, we collected invasion science hypotheses from Daly et al. (2023). We then determined the initial year each hypothesis was proposed based on the publication year of the manuscript, indicating a slowing down (even plateauing) of new hypotheses in recent years (Fig. 1). This is, however, not to say that subsequent studies showing new phenomena or those advancing or testing prior hypotheses are not advances but that the ‘competent’ rethinking has regressed. One such competency demanding rethinking for the field of invasion science could be to bridge the evident disconnect between research and management effectively focused on the ecological dynamics of invasions and studies examining their impacts to enhance the field’s progression, for instance, through the integration of mechanistic biology to foster a cohesive framework that merges the extensive research on the ecology of invasions with the equally significant studies on their impacts.

## Attracting and nurturing competency

Although humans tend to overestimate their own competence (DeAngelis 2003), competency plays a vital role in science, encompassing not only the existing knowledge but also the capability to formulate innovative questions, design novel research approaches, and think creatively. In the ideally ever-evolving realm of (invasion) science, researchers should actively seek out currently unrecognised and uncharted territories. Examples of this include radically new or out-of-the-box ideas, such as stress-testing the definition of non-native species (e.g. should organisms introduced to space via shuttles and space station be considered as non-native species when returning to earth? Sangupta et al. 2024) or exploring the ‘nested’ nature of invasions (non-native species carrying non-native microbiomes influencing invasion success and impacting pathogens and parasites; Holdich et al. 2009). Competency in invasion science should involve domain-specific expertise and the capacity to adapt, innovate, challenge existing assumptions, and bridge gaps between different areas of knowledge as well as governance, social media, and the public (Zeng et al. 2021; Chen et al. 2023). In this dynamic field, fostering competency that embraces creativity and interdisciplinary thinking will be instrumental in uncovering the next frontier of critical questions and driving scientific progress forward.

**Fig. 2:**
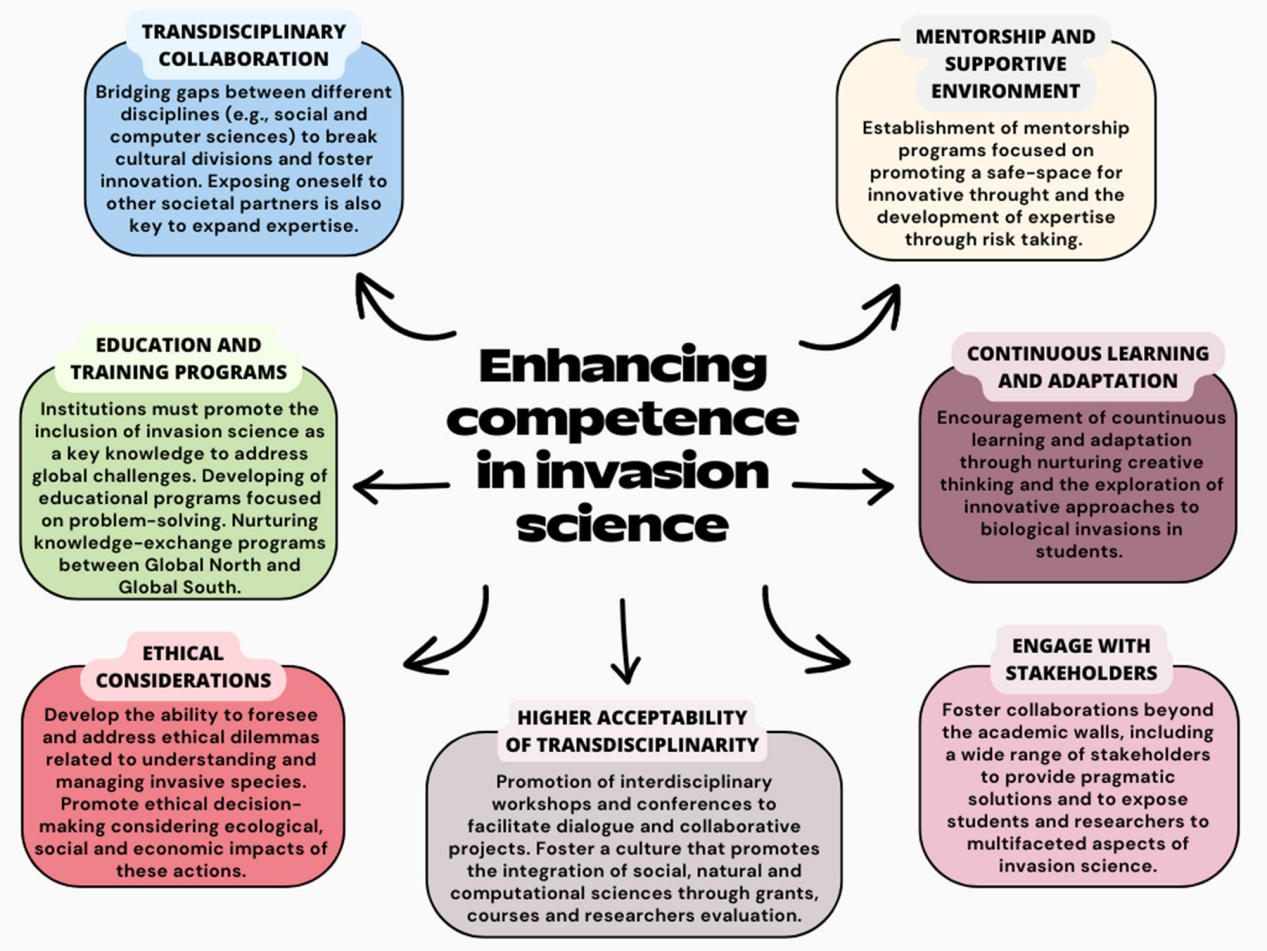
Facets that could facilitate competency in invasion science

Although recent proposals by Ricciardi et al. (2021) are congruent and important steps forward, they could be extended by additional ideas due to the subjective perspectives, backgrounds, and experiences of other authors not included in that specific study, such as standardising the terminology as a basis for a foster growing in the field. While these aspects may not be groundbreaking, implementing and adapting these principles can lead to innovative technological advancements. Nurturing competency, however, requires understanding the factors behind scientific creativity (Yanai and Lercher 2019), namely how we come up with innovative ideas and solutions to old and new problems and how we test or implement them (Van de Ven 1986; Dorst 2015). This is especially difficult in invasion science, as it is essentially a multidisciplinary endeavour, often requiring cross-pollination of ideas and methods across distantly related disciplines. This begets a thorny but needed question: what can we practically do to foster competency moving ahead? Invasion science, as a rapidly evolving discipline, could attract and cultivate competent minds through a multi-faceted approach (Fig. 2):

1. Transdisciplinary collaboration. Given the complexity of biological invasions, we should foster transdisciplinary thinking, especially by bridging gaps between social sciences, which delve into perceptions, misinformation, and denial, and computer sciences, focused on Artificial Intelligence (AI) and big data, thereby breaking cultural divisions across these diverse disciplines. For example, there have been attempts to use AI and big data to predict the spread of invasive species, analyse public perceptions through social media, and support global research networks aimed at integrating diverse scientific insights (Laudy et al. 2020; Lake et al. 2022; Lee et al. 2022). Fostering innovation in this field requires inclusivity of perspectives from diverse disciplines, encouraging individuals not traditionally associated with invasion science to contribute novel insights. However, the target for more pragmatic solutions is to foster collaborations based on transdisciplinarity. To achieve this goal, we may first develop a multidisciplinary playground, we should first and foremost limit discipline-specific jargon in our everyday writing and communication to facilitate exchanges among scholars from distantly related disciplines (Hirst 2003; Martinez and Mammola 2021). At the same time, we should achieve consensus on the meaning of basic technical terms needed for effective communication within invasion science (Hirst 2003). Terminology itself is never static and subject to evolution. On the contrary, it is dynamic and always changing. In invasion science, classifications (often made generally at the species level; Haubrock et al. 2024) have recently been revisited by Soto et al. (2024) to underline the fluid transition between non-native populations that are static or spreading (hence invasive) while acknowledging that impact is a separate dimension pertaining to invasions. After these measures are taken to provide common grounds across disciplines, the transdisciplinarity may be achieved by exposing ourselves to other societal actors that are also involved in dealing with the outcomes of biological invasions (e.g., stakeholders and community groups) to broaden our niche expertise and to co-create solutions that are more integral and practical.
2. Higher acceptability of transdisciplinarity. To enhance the acceptability of transdisciplinary approaches in invasion science, we should promote interdisciplinary workshops and conferences to facilitate dialogue and collaborative projects (Jahn 2008). Funding agencies need to prioritise grants that support transdisciplinary research teams, emphasising the integration of social, natural, and computational sciences (Jaeger-Erben et al. 2008). Universities should develop curricula that incorporate transdisciplinary courses and recognize researchers who engage in such work through awards and promotions. Additionally, online collaborative tools within established platforms like www.ResearchGate.com can help researchers connect and share resources, fostering a culture that values diverse perspectives and comprehensive solutions (Manca 2018). Another crucial recommendation is to promote transdisciplinary thought not only between natural and social sciences but also with the humanities. The focus on outputs, citations, and grants can burden scientists, leading to a mechanisation of approaches, causing scientists to lose some of their humanity and sources of inspiration. Engaging with the humanities can help restore this balance, providing a deeper source of inspiration and a holistic view of scientific endeavours.
3. Education and training programs. Especially considering that invasion science is often not part of the university curriculum, academic institutions and research bodies must emphasise the importance of this field in addressing global ecological challenges, thereby raising its profile and appeal. To nurture competent future scientists, universities and institutes should offer specialised programs and courses that blend theoretical knowledge with practical, hands-on experience in a setting that offers permanency and freedom to unfold and develop their own ideas (Holstermann et al. 2010; Li et al. 2021). This means developing educational programs that focus not only on the core ecological and social aspects of invasion science but also on critical skills such as communication, problem-solving, and ethical considerations, as well as training in methodologies adaptable to different invasion scenarios (Zhang et al. 2019). While international exchange has always been essential, exchanging ideas and people should not be unidirectional. This is, because e.g. attracting students from the Global South and providing funding for their relocation to the Global North can inadvertently lead to a drain of potential from their home regions and the loss of focal knowledge (so-called ‘brain drain’; Sager 2014). This approach does not necessarily contribute to long-term improvements in the Global South, as skills and expertise are needed globally, not just in the economically affluent Global North. Additionally, the attraction of skilled personnel from the Global South risks overlooking, nurturing, and underutilizing existing potential and competencies that are already present in the Global North. Rather, competence and competency must be nurtured everywhere.
4. Ethical considerations. This includes addressing potential biases, considering the welfare of ecosystems and native species, and promoting responsible research practices. Invasion science, while pioneering in its approach to understanding and managing the introduction of non-native species, must confront the ethical challenges inherent in its practices (Tuminello III 2012; Messing 2012). This responsibility entails not only identifying and controlling biological invasions but also a commitment to ethical decision-making that considers the ecological, social, and economic impacts of these actions (Russel 2012; Parke and Russel 2018). Competency is crucial in this context, demanding an innovative and adaptive approach to problem-solving, including the ability to foresee and address ethical dilemmas that currently affect management decisions or have not yet arisen and to develop creative solutions that balance ecological integrity with societal needs through transdisciplinarity. This advanced form of mere competence necessitates a holistic understanding of the multifaceted nature of biological invasion management because ethical issues in invasion science are complex, often involving trade-offs between different ecological goods, such as the preservation of native species versus the control of non-native ones, and socio-economic factors, including the impacts on local communities and economies. Adaptive, creative, and innovative competencies are essential for scientists and practitioners to navigate these complexities effectively.
5. Mentorship and supportive environment. Establish mentorship programs that connect experienced researchers with emerging scholars. It is imperative to cultivate a stable and secure work environment for emerging researchers (Ysseldyk et al. 2019; Dirnagl 2022). This stability is crucial for nurturing new talent and ideas and addressing the prevalent challenge of job insecurity in the scientific sector, which hinders the development of expertise, innovative thought, and competency (Hinds et al. 2000). Create a supportive environment where researchers feel encouraged to take risks, learn from failures, and continuously improve their skills. This also means facilitating collaboration on a global scale. Invasive species often cross borders, and collaboration with researchers from different countries can provide a broader perspective and more comprehensive solutions (Cardoso et al. 2022).
6. Engagement with stakeholders. Beyond academia, we should foster collaborations with a wide range of stakeholders – including government bodies, environmental organisations, and industries – which can provide real-world contexts for research and learning, thus making the field more dynamic and relevant (see e.g. Jekabsone 2019). By engaging in meaningful partnerships, institutions can offer internships, workshops, and collaborative projects that expose students and researchers to the multifaceted aspects of invasion science.
7. Continuous learning and adaptation. Promote a culture of continuous learning and adaptation (Frank and Mohamed 2024), encouraging students and researchers to think beyond traditional boundaries and explore innovative approaches to studying and managing biological invasions (Dorst 2015). Given the dynamic nature of invasion science, staying updated on emerging technologies, methodologies, and global trends is crucial for maintaining continuously growing competences and competencies. Innovation and creative thinking can also be achieved by prioritising project-based learning (Fajra and Novalinda 2020), where students are tasked with developing novel solutions to current challenges (e.g., in invasion science) and participate in courses from other (not necessarily their home) universities. Providing platforms for students to present their ideas, such as conferences or innovation contests, can also stimulate interest and attract talent.

## Conclusion

Just as the Renaissance emerged from the ruins of the Black Plague and the Industrial Revolution from the limitations of agrarian societies, today’s scientific innovations may well be responses to contemporary constraints and challenges. A shift in academic culture is necessary to systematically change and enhance the appeal of (invasion) science. As we have explored, the concept of competency in science transcends mere technical knowledge or academic productivity. Invasion science embodies the ability to innovatively and critically engage with ecological challenges, question established norms, and forge new paths in understanding and managing biological invasions. This multifaceted competency is not just about accumulating data or increasing publication metrics; it is about nurturing a culture of genuine inquiry, cross-disciplinary collaboration, and ethical consideration. Nurturing competency must start at young ages, ideally in schools. At a later stage, developing interdisciplinary research programs and collaborations to tackle complex ecological challenges becomes essential in promoting a holistic understanding of invasion science. Implementing mentorship and training programs that emphasize critical thinking, creativity, and ethical considerations in research can help cultivate the necessary skills and mindset among emerging scientists. Encouraging open-access publications and data sharing fosters global collaboration and transparency, which are vital for the progress of the field. Promoting policies within academic institutions that reward not just publication metrics, but also innovative and impactful research contributions can drive meaningful advancements. Additionally, organizing workshops and conferences focused on the practical application of invasion science to real-world problems can enhance the relevance and impact of the discipline. The advancement of invasion science hence hinges on our capacity to embrace and foster this competency, requiring an inclusive and supportive academic environment and a commitment to continuous learning, ethical research, and global collaboration. As we face increasingly complex environmental challenges, the role of invasion scientists becomes ever more crucial, demanding a blend of creativity, critical thinking, and adaptability. Ultimately, the future of invasion science and its impact on our world depends on our collective ability to cultivate and harness this broader, more dynamic understanding of competency. By doing so, we can ensure that invasion science progresses as a discipline and makes meaningful contributions to addressing the pressing ecological issues of our time.
